# Entropy Generation on Nanofluid Thin Film Flow of Eyring–Powell Fluid with Thermal Radiation and MHD Effect on an Unsteady Porous Stretching Sheet

**DOI:** 10.3390/e20060412

**Published:** 2018-05-28

**Authors:** Mohammad Ishaq, Gohar Ali, Zahir Shah, Saeed Islam, Sher Muhammad

**Affiliations:** 1 Depatment of Mathematics, Islamia College University, Peshawar 25000, Khyber Pakhtunkhwa, Pakistan; 2 Depatment of Mathematics, Abdul Wali Khan University, Mardan 32300, Khyber Pakhtunkhwa, Pakistan; 3 Cecos University of IT and Emerging Sciences Peshawar 25000, Khyber Pakhtunkhwa, Pakistan

**Keywords:** entropy generation, Eyring–Powell fluid, thin film, unsteady porous stretching sheet, nanofluid, HAM, thermal radiation, MHD

## Abstract

This research paper investigates entropy generation analysis on two-dimensional nanofluid film flow of Eyring–Powell fluid with heat amd mass transmission over an unsteady porous stretching sheet in the existence of uniform magnetic field (MHD). The flow of liquid films are taken under the impact of thermal radiation. The basic time dependent equations of heat transfer, momentum and mass transfer are modeled and converted to a system of differential equations by employing appropriate similarity transformation with unsteady dimensionless parameters. Entropy analysis is the main focus in this work and the impact of physical parameters on the entropy profile are discussed in detail. The influence of thermophoresis and Brownian motion has been taken in the nanofluids model. An optima approach has been applied to acquire the solution of modeled problem. The convergence of the HAM (Homotopy Analysis Method) has been presented numerically. The disparity of the Nusslet number, Skin friction, Sherwood number and their influence on the velocity, heat and concentration fields has been scrutinized. Moreover, for comprehension, the physical presentation of the embedded parameters are explored analytically for entropy generation and discussed.

## 1. Introduction

In the past few years, it has been observed that the entropy generation on nanofluid flow analysis has significantly contributed in the area of industries, engineering and technology as well as in other emerging fields of science. Actually, entropy is a thermodynamic quantity representing the unavailability of a system’s thermal energy for conversion into mechanical work, often interpreted as the degree of disorder or randomness in the system. In other words, entropy is a measure of the energy dispersal in the system. We see evidence that the universe tends towards highest entropy many places in our lives. A campfire is an example of entropy. The solid wood burns and becomes ash, smoke and gases, all of which spread energy outwards more easily than the solid fuel. Thin film flow problems have diverse applications in many fields, fluctuating from specific situations in the flow in human lungs to lubrication problems in engineering, which is probably one of the largest subfields of thin film flow problems. The practical applications of thin film flow is a challenging interplay between fluid mechanics and structural mechanics. A nanofluid is comprised of a base fluid with tiny (nanometer) sized nanoparticles, such as carbides or carbon nanotubes, oxides, and metals, whereas traditional base liquids involve ethylene glycol, oil, and water. A nanofluid is very helpful in enhancing thermal conductivity and convection of heat transfer coefficient when it is analyzed with the base fluid. In modern technology, nanomaterials are becoming increasingly important in the performance of various heat exchangers, such as microelectronics, optical modulators, and chemical production. Magneto-nanofluids are also remarkable for their use in various applications, such as tunable optical fiber filters, magneto-optical wavelength filters, optical modulators, and optical switches. In biomedical engineering, magneto-nanoparticles are also very helpful in cancer therapy, sink-float separation, hyperthermia, magnetic resonance imaging (MRI), magnetic cell separation, drug delivery, and magnetic drug targeting. In particular, heat transfer and convective flow are influenced by the features of nanofluids, such as thermal conductivity and viscosity. Conventional heat transfer in various Newtonian and non-Newtonian fluids, such as ethylene glycol, oil, water, etc. holds a poor rate of heat transfer. However, the thermal conductivity of these kinds of fluids plays a significant role in the heat transfer coefficient between a heat transfer surface and heat transfer medium. During the last few decades, an innovative methodology has been used to enhance heat transfer with the help of ultra-fine solid particles in fluids. Non-Newtonian fluids are of various kinds; thus, all non-Newtonian fluids cannot be addressed by using single constitutive expression between stress and shear rate. Much attention has been devoted to study different models of non-Newtonian fluids. Among them is an Eyring–Powell fluid model, which is very complex, but it has certain advantages over other fluid models. Firstly, it was derived from a kinetic theory of liquids rather than the empirical relation as in the case of the Power-law model. Secondly, it appropriately reduces to Newtonian behavior for low and high shear rates. The Eyring–Powell fluid model describes properties of shear thinning fluids. Examples of such fluids are human blood, ketchup, toothpaste, etc. Thus, researchers are attracted to investigate its thermo physical properties. In view of all these applications, it becomes an important issue for researchers to develop the study of liquid film flow on the stretching sheet. The flow of liquid film was first studied for viscous flow and further it is extended to non-Newtonian fluids. Crane [1] was the first one who deliberated the motion of viscous fluid in a linear stretching surface. Dandapat [2] studied the flow of viscoelastic fluids having a heat transfer on a stretching sheet. Wang [3] was the pioneer to investigate unsteady stretching surface and finite liquid film flow on it. Usha and Sridharan [4] have worked on the same problem and extended it to liquid film fluid with heat transmission analysis on horizontal sheet. Liu and Andersson [5] have used numerical techniques in their work to obtain solutions and discussed parameters. Aziz et al. [6] observed the effect of inner temperature production in an unsteady stretching sheet due to flow in a thin liquid film on it. Tawade et al. [7] inquired the flow of thin fluid on an unsteady porous surface having thermal radiation, in the existence of magnetic field, applying Newton Raphson and Runge–Kutta Fehlberg formulae for solutions of nonlinear equations. A brief discussion is given on physical parameters in his work too. Thin film flow of non-Newtonian fluids are in abundance in many walks of life. Therefore, it is one of the most common factors of the nature which is mostly used in the field of industry , engineering and technology. Andersson [8] was the pioneer to study flow of thin liquid film of non-Newtonian fluids by taking into account the Power Law model in an unsteady stretching sheet. After that, most of the researchers [9,10,11,12] have investigated the Power Law of fluid using different cases in an unsteady stretching sheet. Megahed et al. [13] observed thin film flow of Casson fluid and temperature transmission in existance of viscous dissipation and variable heat flux with slip velocity. Abolbashari et al. [14] explored the same fluid with entropy generation with nano particles. Recently, Qasim et al. [15] applied Buongiorno’s model to study thin film of the nanofluid taking an unsteady porous sheet. Eyring–Powell fluid is an integral part of non-Newtonian fluids. Many researchers investigated the effect of MHD and heat on Eyring–Powell fluid. The at hand amount of study in the form of nanofluids is less than the least amount. Hayat et al. [16] derived Eyring–Powell fluid model from kinetic theory of liquids instead of empirical relation. Sirohi et al. [17] have reported some studies on flows of Eyring–Powell fluid. Eldabe et al. [18] modeled Eyring–Powell fluid of the thermal radiation’s impact on the Megnatohydrodynamics 3D flow. Patel et al. [19] applied method of satisfaction with asymptotic boundary conditions for numerical solution of Eyring–Powell fluid flow. The survey of temperature transfer with entropy generation has been explored by many scientists [20,21,22]. Thermodynamic systems have involved a lot of processes, which includes entropy generation such as diffusion, friction and viscosity. In fields of turbomachinery, heat exchangers and electronic cooling entropy generation have received an extraordinary curiosity. In particular, some sort of heat irreversibility is involved in every thermal process due to the existence of temperature gradient. It reduces the quality of energy and administers a measured deficiency. As far as the recent research in thermodynamics is concerned, the thermodynamics’ 2nd law [23] is comparatively more suitable and well organized in optimizing a system than that of the thermodynamics’ 1st law. The essential logic distinguishing these laws is the analysis on the thermodynamics’ 1st law, reflecting that it does not furnish energy variation but only employ energy description. Recently, the influence of irreversibility on the interplay of energy has collected great recognition. For example, Rashidi et al. [24] analytically investigated entropy generation of a nanofluid in a steady flow over a porous rotating disk employing MHD. Qing et al. [25] have studied entropy generation on the Casson nanofluid over the shrinking sheet under the impact of MHD. Entropy generation has also been explored with detail in [26,27,28,29,30]. In the field of science and technology, most of the mathematical problems are complex in their nature and the exact solution is almost very difficult or at times impossible. Numerical and Analytical methods are used to find out the approximate solution of such problems. One of the popular and proficient methods to solve such types of problems is the Homotopy Analysis Method. Its main advantage is that it is applicable to the nonlinear differential equations without discretization or linearization and is a substitute method. Liao [31,32,33,34,35,36,37,38] was the first one to investigate this technique to solve problems and generally verified that this method converges rapidly to the approximate solutions. This method also provides series solutions that include single variable functions. The significance of this method is that it takes into account all parameters involved in the problem. The behavior of these physical parameters can be easily explored. Due to its fast convergence, many researchers like Rashidi [39,40], Abbasbandy [41,42,43], Hayat et al. [44,45], and Nadeem et al. [46,47] used this technique to solve highly nonlinear and coupled equations. Recently, Xiao et al. [48] has applied a novel method “Fractal-Monte Carlo Technique” on heat and mass transfer of porous media including porous stretching sheet in his research article, “Research on Relative Permeability of Nanofibers with Capillary Pressure Effect by Means of Fractal-Monte Carlo Technique”. Recently, Shah et al. [49,50] studied the effects of hall current on three dimensional non-Newtonian nanofluids and micropolar nanofluids in a rotating frame. To the best of our knowledge, no such attempt has been made on studying entropy generation of an MHD Eyring–Powell fluid through a permeable stretching sheet. With motivation from the above analysis in mind, the aim of the present study was to analyze the entropy generation on nanofluid thin film flow of Eyring–Powell fluid with thermal radiation and MHD effect on an unsteady porous stretching surface. The governing flow problem is comprised of the momentum equation, energy equation, and nanoparticle concentration equation, which are further transformed into ordinary differential equations using similarity transformation variables. The reduced ordinary coupled differential equations are solved numerically with the help of the Homotopy Analysis Method (HAM).

## 2. Basic Equations

The equation of continuity is

(1) $$div\hat{V}=0,$$

The equation of momentum is

(2) $$\rho a_{i}=-\nabla p+\nabla \left(T\right)+\hat{J}\times \hat{B},$$

The equation of heat transfer is

(3) $$\left(\hat{V}.\nabla \right)T=\alpha \nabla^{2}T+\rho \left[\begin{array}{c} D_{B}\nabla C.\nabla T+\frac{D_{T}}{T_{0}}\nabla T.\nabla T \end{array}\right],$$

The equation of mass transfer is

(4) $$\left(\hat{V}.\nabla \right)C=D_{B}\nabla^{2}C+\frac{D_{T}}{T_{0}}\nabla^{2}T.$$

The constitutive equation for a Cauchy stress in an Eyring–Powell model fluid [37] is given by

(5) $$\tau_{ij}=\mu \frac{\partial u_{i}}{\partial x_{j}}+\frac{1}{\beta^{*}}\sinh^{-1}\left[\begin{array}{c} \frac{1}{c}\frac{\partial u_{i}}{\partial x_{j}} \end{array}\right],$$

(6) $$\sinh^{-1}\left[\begin{array}{c} \frac{1}{c}\frac{\partial u_{i}}{\partial x_{j}} \end{array}\right]\approx \frac{1}{c}\frac{\partial u_{i}}{\partial x_{j}}-\frac{1}{6}{\left[\begin{array}{c} \frac{1}{c}\frac{\partial u_{i}}{\partial x_{j}} \end{array}\right]}^{3},\left|\frac{1}{c}\frac{\partial u_{i}}{\partial x_{j}}\right|≺1,$$

where $u_{i}$ is the velocity, $\tau_{ij}$ is the Cauchy stress tensor, μ is the coefficient of shear vescosity and $\beta^{*}$ and c represents characteristics of the Eyring–Powell fluid.

## 3. Mathematical Formulation

Assume two-dimensional incompressible nanofluid liquid film flow of Eyring–Powell fluid having thermal radiation on the unsteady porous stretching surface with simultaneous transfer of mass and heat. The coordinate axes are selected such that the slit is along the direction of *x*-axis and surface is perpendicular to *y*-axis. Linear velocity of the plate is along positive *x*-axis and assumed as

(7) $$U_{0}\left(x,t\right)=\frac{\alpha x}{1-\gamma t},$$

which is stretching, where γ is the stretching parameter. The surface temperature of the nanofluid is

(8) $$T_{w}\left(x,t\right)=T_{0}-T_{ref}\left(\begin{array}{c} \frac{\alpha x^{2}}{2v} \end{array}\right)\times {\left(1-\gamma t\right)}^{-3/2},$$

and similarly the volume concentration for the nanofluid is

(9) $$C_{w}\left(x,t\right)=C_{0}-C_{ref}\left(\begin{array}{c} \frac{\alpha x^{2}}{2v} \end{array}\right)\times {\left(1-\gamma t\right)}^{-3/2}.$$

The time dependent term $\frac{\alpha x^{2}}{v(1-\gamma t)}$ can be documented as the local Reynold number, dependent on the stretching velocity $U_{0}\left(x,t\right)$. where $T_{0}$, $C_{0}$ is defined as temperature and concentration at the slit respectively, $C_{ref}$ and $T_{ref}$ are the reference concentration and reference heat such that $C_{ref}\in \left[0,C_{0}\right]$ and $T_{ref}\in \left[0,T_{0}\right]$. At the start, the slit is initiated along the Origin and after that external force is acted to stretch the slit in the positive *x*-axis direction at the rate $\frac{\alpha }{1-\gamma t}$ in the time γ∈[0,1] with $U_{0}\left(x,t\right)$ initial velocity.

The basic governing equations are as follows [16]:

Considering the above assumptions, the leading equations of continuity, momentum, energy and concentration of two-dimensional thin film flow are as under [19]

(10) $$\frac{\partial \hat{u}}{\partial x}+\frac{\partial \hat{v}}{\partial y}=0,$$

(11) $$\frac{\partial \hat{u}}{\partial t}+\hat{u}\frac{\partial \hat{u}}{\partial x}+\hat{v}\frac{\partial \hat{u}}{\partial y}=\left(\begin{array}{c} v+\frac{1}{\rho \beta C} \end{array}\right)\frac{\partial^{2}\hat{u}}{\partial y^{2}}-\frac{1}{2\rho \beta C^{3}}\left[\begin{array}{c} {\left(\begin{array}{c} \frac{\partial \hat{u}}{\partial y} \end{array}\right)}^{2}\frac{\partial^{2}\hat{u}}{\partial y^{2}} \end{array}\right]-\frac{\sigma {\hat{B}}_{0}^{2}}{\rho }\hat{u}\left(t\right)-\frac{\nu }{\rho K}\hat{u}\left(t\right),$$

(12) $$\frac{\partial T}{\partial t}+\hat{u}\frac{\partial T}{\partial x}+\hat{v}\frac{\partial T}{\partial y}=\frac{1}{\rho c_{p}}\frac{\partial }{\partial y}\left[\begin{array}{c} k_{p}\frac{\partial T}{\partial y} \end{array}\right]+t\left[\begin{array}{c} D_{B}\left(\begin{array}{c} \frac{\partial C}{\partial y}\frac{\partial T}{\partial y} \end{array}\right)+\frac{D_{t}}{T_{0}}{\left(\begin{array}{c} \frac{\partial T}{\partial y} \end{array}\right)}^{2} \end{array}\right]-\frac{1}{\rho c_{p}}\frac{\partial q_{r}}{\partial y},$$

(13) $$\frac{\partial C}{\partial t}+\hat{u}\frac{\partial C}{\partial x}+\hat{v}\frac{\partial C}{\partial y}=D_{B}\frac{\partial^{2}C}{\partial y^{2}}+\left(\begin{array}{c} \frac{D_{T}}{T_{0}} \end{array}\right)\frac{\partial^{2}T}{\partial y^{2}}.$$

Here, $\hat{u}$ and $\hat{v}$ represent the components of fluid velocity, υ denotes the coefficient of kinematic viscosity, ρ represents density where as σ and μ represent the electrical conductivity and dynamic viscosity respectively, T represents the temperature, α is thermal diffusivity, *K* represents porosity, $c_{p}$ represents specific heat, thermal conductivity of fluid is represented by $k_{p}$, Brownian diffusion coefficient is denoted by $D_{B}$, $t=\frac{{\left(\rho c_{p}\right)}_{p}}{{\left(\rho c_{p}\right)}_{f}}$, where $\rho_{f}$ denotes the base fluid density and ρ represents density of particle, and C is the coefficient of volumetric expansion. $q_{r}$ indicates the radioactive heat fluctuation, which is given by Rosseland approximation as

(14) $$q_{r}=-\frac{16\sigma^{*}}{3\kappa^{*}}\frac{\partial T^{4}}{\partial y},$$

where $\kappa^{*}$, $\sigma^{*}$ denoted the mean absorption coefficient and the Stefan Boltzmann constant, respectively. Applying Taylor’s series, we have

(15) $$T^{4}=T_{0}^{4}+4T_{0}^{3}{\left(\begin{array}{c} T-T_{0} \end{array}\right)}^{2}+\ldots$$

By ignoring the higher order term, we obtain

(16) $$T^{4}=4TT_{0}^{3}-3T_{0}^{4}.$$

Now, using Equation (16) in Equation (14), the term $\frac{\partial q_{r}}{\partial y}$ reduced to the form of

(17) $$\frac{\partial q_{r}}{\partial y}=-\frac{16\sigma T_{0}^{3}}{3\kappa^{*}}\frac{\partial^{2}T}{\partial y^{2}}.$$

The selected Boundary conditions are

(18) $$\hat{u}=U_{0},\;\hat{v}=0,\;T=T_{w},\;C=C_{s}\;at\;y=0,$$

(19) $$\frac{\partial \hat{u}}{\partial y}=\frac{\partial T}{\partial y}=\frac{\partial C}{\partial y}=0,\;\hat{v}=\frac{dh}{dx}=0,at\;y=h\left(t\right),$$

where the thickness of liquid is h(t). The similarity variables for non-dimensionalization are as follows:

(20) $$\begin{array}{c} \eta =\sqrt{\frac{\alpha }{\upsilon (1-\gamma t)}}y,\;\Psi \left(x,y,t\right)=x\sqrt{\frac{\upsilon \alpha }{1-\gamma t}}f\left(\eta \right),\\ T\left(x,y,t\right)=T_{0}-T_{ref}\left(\begin{array}{c} \frac{\alpha x^{2}}{2v} \end{array}\right){\left(1-\gamma t\right)}^{-3/2}\theta \left(\eta \right),\\ C\left(x,y,t\right)=C_{0}-C_{ref}\left(\begin{array}{c} \frac{\alpha x^{2}}{2v} \end{array}\right){\left(1-\gamma t\right)}^{-3/2}\phi \left(\eta \right). \end{array}$$

Here, Ψ is stream function such that $\left(\begin{array}{c} \hat{u},\hat{v} \end{array}\right)=\left(\begin{array}{c} \frac{\partial \Psi }{\partial y},-\frac{\partial \Psi }{\partial x} \end{array}\right)$. The prime indicates derivative w.r.t η and the thickness of non-dimensional nanofluid film is represented by β, where $\beta =\left[\begin{array}{c} \frac{\alpha }{\upsilon (1-\gamma t)} \end{array}\right]h\left(t\right)$:

(21) $$\frac{dh}{dt}=-\frac{\beta \gamma }{2}{\left[\begin{array}{c} \frac{\upsilon }{\alpha } \end{array}\right]}^{\frac{1}{2}}{\left(\begin{array}{c} 1-\gamma t \end{array}\right)}^{-\frac{1}{2}}.$$

Inserting Equations in (20) in the Equations (10)–(13), where Equation (10) identically holds and we get the following governing equations:

(22) $$\left(1+k\right)f^{\prime \prime \prime }-{\left(f^{\prime }\right)}^{2}+ff^{\prime \prime }-A\left(\begin{array}{c} f^{\prime }+\frac{\eta }{2}f^{\prime \prime } \end{array}\right)-\xi {\left(f^{\prime \prime }\right)}^{2}f^{\prime \prime \prime }-k^{*}f^{\prime }-Mf^{\prime }=0,$$

(23) $$\left[\begin{array}{c} 1+\frac{4}{3}Rd \end{array}\right]\theta^{\prime \prime }+Pr\left[\begin{array}{c} f\theta^{\prime }-2f^{\prime }\theta -\frac{A}{2}\left(\begin{array}{c} 3\theta +\eta \theta^{\prime } \end{array}\right)+Nb\phi^{\prime }\theta^{\prime }+Nt{\left(\theta^{\prime }\right)}^{2} \end{array}\right]=0,$$

(24) $$\phi^{\prime \prime }+Sc\left[\begin{array}{c} f\phi^{\prime }-2f^{\prime }\phi -\frac{A}{2}\left(\begin{array}{c} 3\phi +\eta \phi^{\prime \prime } \end{array}\right) \end{array}\right]+\frac{N_{t}}{N_{b}}\phi^{\prime \prime }=0.$$

The corresponding boundary conditions are

(25) $$f^{\prime }\left(0\right)=1,\;f\left(0\right)=0,\;\theta \left(0\right)=\phi \left(0\right)=1,$$

(26) $$f\left(\beta \right)=\frac{A\beta }{2},\;f^{\prime \prime }\left(\beta \right)=0,\;\theta^{\prime }\left(\beta \right)=\phi^{\prime }\left(\beta \right)=0.$$

The non-dimensional parameters after simplification can be defined as $Pr=\frac{v}{\alpha }$ is prandtl number, $Rd=\frac{4\sigma^{*}T_{s}^{3}}{{\left(\rho c_{p}\right)}_{f}\kappa k_{p}}$, is radiation parameter, $M=\frac{\sigma B_{0}^{2}}{\rho \alpha }\left(1-\gamma t\right)$ is magnetic parameter, $A=\frac{\gamma }{\alpha }$ is unsteadiness parameter and $Sc=\frac{v}{D_{B}}$ Schmid number, $Nb=\frac{\tau D_{B}\left(C_{\omega }-C_{0}\right)}{v}$ is parameter of Brownian motion, $Nt=\frac{{\left(\rho c_{p}\right)}_{p}D_{T}T_{0}}{{\left(\rho c_{p}\right)}_{f}T_{c}}$ is Thermophoretic parameter, $\xi =\frac{1}{2\rho \beta C^{3}}{\left(\begin{array}{c} \frac{\alpha }{1-\gamma t} \end{array}\right)}^{3}{\left(\begin{array}{c} \frac{x}{r} \end{array}\right)}^{3}$ is Stretching parameter, $k^{*}=\frac{\upsilon }{\rho K}\left(\begin{array}{c} 1-\gamma t \end{array}\right)$ is Porosity parameter, and $k=\frac{1}{\mu BCr}$ is Eyring–Powell fluid parameter.

## 4. Physical Quantities

For our physical interest, the Skin Friction is defined as ${\hat{C}}_{f}=\frac{{\left({\hat{S}}_{xy}\right)}_{y=0}}{\rho {\hat{u}}_{w}^{2}}$, the Nusselt number is defined $Nu=\frac{hQ_{w}}{\hat{k}\left(T_{0}-T_{h}\right)}$,$Q_{w}$ is heat flux and $Q_{w}=-\hat{k}{\left(\begin{array}{c} \frac{\partial T}{\partial y} \end{array}\right)}_{y=0}$ and the Sherwood number is defined as $Sh=\frac{hJ_{w}}{D_{B}\left(C_{0}-C_{h}\right)}$, $J_{w}$ is mass flux and $J_{w}=-D_{B}{\left(\begin{array}{c} \frac{\partial C}{\partial y} \end{array}\right)}_{y=0}$.

The dimensionless form of $C_{f}$, Nu and Sh are obtained as:

(27) $$C_{f}=\left(1+k\right)f^{\prime \prime }\left(0\right),Nu=\left(\begin{array}{c} 1+\frac{4}{3}\theta^{\prime \prime }\left(0\right) \end{array}\right),Sh=-\phi^{\prime }\left(0\right).$$

## 5. Solution of the Problem by Homotopy Analysis Method

Liao in 1992 was the first one who proposed the Homotopy analysis method. He deduced HAM from one of the fundamental ideas of the topology called Homotopy. He used two Homotopic functions in the derivation of this technique. The functions are called to be Homotopic when one function can be continuously distorted into another. HAM is a substitute method and its main importance is that it is applied to the nonlinear differential equations without discretization and linearization. This technique has several advantages, some of them are (a) It is free from the values of the parameters which may be small or large. (b) Its declarations about the convergence of the solution. (c) It is independent from collection of base function and linear operator. The solution of Equations (22)–(24), with the reliable boundary conditions (25) and (26), are gained by use of analytic method. Solution results obtained by HAM contained the assisting parameters which regulate and control to converge the solutions and bases functions. The initial guesses are

(28) $$\hat{f_{0}}\left(\eta \right)=\eta ,\;\theta_{0}\left(\eta \right)=1,and\;\phi_{0}\left(\eta \right)=1.$$

$L_{f}$, $L_{\theta }$, and $L_{\phi }$ are representing linear operators

(29) $$L_{f}\left(\hat{f}\right)=\hat{f^{\prime \prime \prime }},\;L_{\theta }\left(\theta \right)=\theta^{\prime \prime },\;L_{\phi }\left(\phi \right)=\phi^{\prime \prime },$$

which have the subsequent application

(30) $$L_{f}\left(e_{1}+e_{2}\eta +e_{3}\eta^{2}\right)=0,L_{\theta }\left(e_{4}+e_{5}\eta \right)=0,L_{\phi }\left(e_{6}+e_{7}\eta \right)=0.$$

The coefficients involve in the general solution are $e_{i}$, where 1≤i≤7. The corresponding nonlinear operators $N_{f}$, $N_{\theta }$, $N_{\phi }$ are carefully chosen of the form:

(31) $$N_{f}\left[\begin{array}{c} \hat{f}\left(\eta ;\xi \right) \end{array}\right]=\left(1+k\right)\frac{\partial^{3}\hat{f}}{\partial \eta^{3}}-{\left(\begin{array}{c} \frac{\partial \hat{f}}{\partial \eta } \end{array}\right)}^{2}+f\frac{\partial^{2}\hat{f}}{\partial \eta^{2}}-A\left(\begin{array}{c} \frac{\partial \hat{f}}{\partial \eta }+\frac{\eta }{2}\frac{\partial^{2}\hat{f}}{\partial \eta^{2}} \end{array}\right)-\lambda {\left(\begin{array}{c} \frac{\partial^{2}\hat{f}}{\partial \eta^{2}} \end{array}\right)}^{2}\frac{\partial^{3}\hat{f}}{\partial \eta^{3}}-k^{*}\frac{\partial \hat{f}}{\partial \eta }-M\frac{\partial \hat{f}}{\partial \eta },$$

(32) $$N_{\theta }\left[\begin{array}{c} \hat{f}\left(\eta ;\xi \right),\theta \left(\eta ;\xi \right),\phi \left(\eta ;\xi \right) \end{array}\right]=\left(\begin{array}{c} 1+\frac{4}{3}Rd \end{array}\right)\frac{\partial^{2}\theta }{\partial \eta^{2}}+Pr\left[\begin{array}{c} \hat{f}\frac{\partial \theta }{\partial \eta }-2\theta \frac{\partial \hat{f}}{\partial \eta }-\frac{A}{2}\left(\begin{array}{c} 3\theta +\eta \frac{\partial \theta }{\partial \eta } \end{array}\right)+Nt{\left(\begin{array}{c} \frac{\partial \theta }{\partial \eta } \end{array}\right)}^{2}\\ +Nb\frac{\partial \theta }{\partial \eta }\frac{\partial \phi }{\partial \eta } \end{array}\right],$$

(33) $$N_{\phi }\left[\begin{array}{c} \hat{f}\left(\eta ;\xi \right),\theta \left(\eta ;\xi \right),\phi \left(\eta ;\xi \right) \end{array}\right]=\frac{\partial^{2}\phi }{\partial \eta^{2}}+Sc\left[\begin{array}{c} \hat{f}\frac{\partial \phi }{\partial \eta }-2\phi \frac{\partial \hat{f}}{\partial \eta }-\frac{A}{2}\left(\begin{array}{c} 3\phi +\eta \frac{\partial^{2}\phi }{\partial \eta^{2}} \end{array}\right) \end{array}\right]+\frac{Nt}{Nb}\frac{\partial^{2}\theta }{\partial \eta^{2}}.$$

The elementary solution procedure by HAM is defined in Equations (28)–(56).

### 5.1. Equations of Zeroth-Order Deformation

The 0^*th*^ Order system form Equations (34)–(36)

(34) $$\left(1-\zeta \right)L_{f}\left[\hat{f}\left(\eta ,\zeta \right)-{\hat{f}}_{0}\left(\eta \right)\right]=ph_{f}N_{f}\left[\hat{f}\left(\eta ,\zeta \right)\right],$$

(35) $$\left(1-\zeta \right)L_{\theta }\left[\theta \left(\eta ,\zeta \right)-\theta_{0}\left(\eta \right)\right]=ph_{\theta }N_{\theta }\left[\hat{f}\left(\eta ,\zeta \right),\theta \left(\eta ,\zeta \right),\phi \left(\eta ,\zeta \right)\right],$$

(36) $$\left(1-\zeta \right)L_{\phi }\left[\phi \left(\eta ,\zeta \right)-\phi_{0}\left(\eta \right)\right]=\zeta h_{\phi }N_{\phi }\left[\hat{f}\left(\eta ,\zeta \right),\theta \left(\eta ,\zeta \right),\phi \left(\eta ,\zeta \right)\right].$$

The corresponding boundary constraints are

(37) $$\hat{f}\left(\eta ,\zeta \right){{|}_{\eta =0}=0,\;\hat{f}\left(\eta ,\zeta \right)|}_{\eta =\beta }=\frac{A\beta }{2},$$

(38) $$\frac{\partial \hat{f}\left(\eta ,\zeta \right)}{\partial \eta }{{|}_{\eta =0}=1,\;\frac{\partial^{2}\hat{f}\left(\eta ,\zeta \right)}{\partial \eta^{2}}|}_{\eta =\beta }=0,$$

(39) $${\theta \left(\eta ,\zeta \right)|}_{\eta =0}=1,\;\frac{\partial \theta (\eta ,\zeta )}{\partial \eta }{|}_{\eta =\beta }=0,$$

(40) $${\phi \left(\eta ,\zeta \right)|}_{\eta =0}=1,\;\frac{\partial \phi (\eta ,\zeta )}{\partial \eta }{|}_{\eta =\beta }=0,$$

where ζ∈[0,1] is the embedding constraint, and $h_{f}$, $h_{\theta }$, $h_{\phi }$ are used to regulate convergence. When ζ=0 and ζ=1, we obtain:

(41) $$\hat{f}\left(\eta \right)=\hat{f}\left(\eta ,1\right),\;\theta \left(\eta \right)=\theta \left(\eta ,1\right),\;\phi \left(\eta \right)=\phi \left(\eta ,1\right).$$

Taylor’s series approximation for ζ=0 is used to expand the velocity, temperature and concentration fields $\hat{f}\left(\eta ,\zeta \right),\theta \left(\eta ,\zeta \right)$, and ϕ(η,ζ)

(42) $$\hat{f}\left(\eta ,\zeta \right)={\hat{f}}_{0}\left(\eta \right)+\sum_{k=1}^{\infty }{\hat{f}}_{k}\left(\eta \right)\zeta^{k},$$

(43) $$\theta \left(\eta ,\zeta \right)=\theta_{0}\left(\eta \right)+\sum_{k=1}^{\infty }\theta_{k}\left(\eta \right)\zeta^{k},$$

(44) $$\phi \left(\eta ,\zeta \right)=\phi_{0}\left(\eta \right)+\sum_{k=1}^{\infty }\phi_{k}\left(\eta \right)\zeta^{k},$$

(45) $${\hat{f}}_{n}\left(\eta \right)=\frac{1}{n!}\frac{\partial \hat{f}\left(\eta ,\zeta \right)}{\partial \eta }{|}_{\zeta =0},\;\theta_{n}\left(\eta \right)=\frac{1}{n!}\frac{\partial \theta (\eta ,\zeta )}{\partial \eta }{|}_{\zeta =0},\;\phi_{n}\left(\eta \right)=\frac{1}{n!}\frac{\partial \phi (\eta ,\zeta )}{\partial \eta }{|}_{\zeta =0}.$$

The secondary restrictions $h_{f}$, $h_{\theta }$ and $h_{\phi }$ are chosen in a manner that the series (42)–(44) converges at ζ=1 so, switching ζ=1 in (42)–(44), we obtain:

(46) $$\hat{f}\left(\eta \right)={\hat{f}}_{0}\left(\eta \right)+\sum_{n=1}^{\infty }{\hat{f}}_{n}\left(\eta \right),$$

(47) $$\theta \left(\eta \right)=\theta_{0}\left(\eta \right)+\sum_{n=1}^{\infty }\theta_{n}\left(\eta \right),$$

(48) $$\phi \left(\eta \right)=\phi_{0}\left(\eta \right)+\sum_{n=1}^{\infty }\phi_{n}\left(\eta \right).$$

### 5.2. Equation of the n^th^ Order Deformation

The $n^{th}$ order problem satisfies the following:

(49) $$L_{f}\left[\begin{array}{c} {\hat{f}}_{n}\left(\eta \right)-\chi_{n}{\hat{f}}_{n-1}\left(\eta \right) \end{array}\right]=h_{f}R_{k}^{f}\left(\eta \right),$$

(50) $$L_{\theta }\left[\begin{array}{c} \theta_{n}\left(\eta \right)-\chi_{n}\theta_{n-1}\left(\eta \right) \end{array}\right]=h_{\theta }R_{n}^{\theta }\left(\eta \right),$$

(51) $$L_{\phi }\left[\begin{array}{c} \phi_{n}\left(\eta \right)-\chi_{n}\phi_{n-1}\left(\eta \right) \end{array}\right]=h_{\phi }R_{n}^{\phi }\left(\eta \right).$$

The consistent boundary conditions are:

(52) $${\hat{f}}_{n}\left(0\right)={\hat{f}}_{n}^{\prime }\left(0\right)=\theta_{n}\left(0\right)=\phi_{n}\left(0\right)=0,\;{\hat{f}}_{n}^{\prime \prime }\left(\beta \right)=\theta_{n}^{\prime }\left(\beta \right)=\phi_{n}^{\prime }\left(\beta \right)=0.$$

Here,

(53) $$\begin{array}{c} R_{n}^{f}\left(\eta \right)=\left(1+k\right){\hat{f}}_{n-1}^{\prime \prime \prime }-\sum_{k=0}^{n-1}{\hat{f}}_{n-1-k}^{\prime }{\hat{f}}_{k}^{\prime }+\sum_{k=0}^{n-1}{\hat{f}}_{n-1-k}{\hat{f}}_{k}^{\prime \prime }-A\left[\begin{array}{c} {\hat{f}}_{n-1}^{\prime }+\frac{\eta }{2}{\hat{f}}_{n-1}^{\prime \prime } \end{array}\right]\\ +\lambda \sum_{k=0}^{n-1}{\left(\begin{array}{c} {\hat{f}}_{n-1-k}^{\prime \prime } \end{array}\right)}^{2}{\hat{f}}_{k}^{\prime \prime \prime }-M{\hat{f}}_{n-1}^{\prime }-k^{*}{\hat{f}}_{n-1}^{\prime }, \end{array}$$

(54) $$R_{n}^{\theta }\left(\eta \right)=\left(\begin{array}{c} 1+\frac{4}{3}Rd \end{array}\right)\theta_{n-1}^{\prime \prime }+Pr\left[\begin{array}{c} \sum_{k=0}^{n-1}{\hat{f}}_{n-1-k}\theta_{k}^{\prime }-2\sum_{k=0}^{n-1}{\hat{f}}_{n-1-k}^{\prime }\theta_{k}-\frac{A}{2}\left(\begin{array}{c} 3\theta_{n-1}+\eta \theta_{n-1}^{\prime } \end{array}\right)\\ +\sum_{k=0}^{n-1}\theta_{n-1-k}\theta_{k}^{\prime \prime }+Nb\sum_{k=1}^{n-1}\theta_{n-1-k}^{\prime }\phi_{k}^{\prime }+Nt\sum_{k=1}^{n-1}\theta_{n-1-k}^{\prime }\theta_{k}^{\prime } \end{array}\right],$$

(55) $$R_{n}^{\phi }\left(\eta \right)=\phi_{n-1}^{\prime \prime }+Sc\left[\begin{array}{c} \sum_{k=0}^{n-1}{\hat{f}}_{n-1-k}\phi_{k}^{\prime }-2\sum_{k=0}^{n-1}{\hat{f}}_{n-1-k}^{\prime }\phi_{k}-\frac{A}{2}\left(\begin{array}{c} 3\phi_{n-1}+\eta \phi_{n-1}^{\prime } \end{array}\right) \end{array}\right]+\frac{Nt}{Nb}\theta_{n-1}^{\prime \prime },$$

where

(56) $$\chi_{n}=\left\{\begin{array}{cc} 0, & \zeta \;<\;1,\\ 1, & \zeta \;>\;1. \end{array}\right.$$

## 6. Entropy Generation Analysis

Entropy generation(volumetric) for the Eyring–Powell fluid is as follows:

(57) $$\begin{array}{c} S_{gen}^{\prime \prime \prime }=\frac{k}{T_{0}^{2}}\left[\begin{array}{c} {\left(\begin{array}{c} \frac{\partial T}{\partial y} \end{array}\right)}^{2}+\frac{16\bar{\sigma }T^{3}}{3\bar{k}}{\left(\begin{array}{c} \frac{\partial T}{\partial y} \end{array}\right)}^{2} \end{array}\right]+\frac{\mu }{T_{0}}\left[\begin{array}{c} \left(\begin{array}{c} 1+\frac{1}{\rho BC} \end{array}\right){\left(\begin{array}{c} \frac{\partial \hat{u}}{\partial y} \end{array}\right)}^{2}-\frac{1}{6\rho BC}{\left(\begin{array}{c} \frac{\partial \hat{u}}{\partial y} \end{array}\right)}^{4} \end{array}\right]+\\ \frac{RD}{C_{0}}{\left(\begin{array}{c} \frac{\partial C}{\partial y} \end{array}\right)}^{2}+\frac{\sigma B_{0}^{2}}{T_{0}}{\hat{u}}^{2}+\frac{RD}{T_{0}}\left(\begin{array}{c} \frac{\partial T}{\partial y}\frac{\partial C}{\partial y}+\frac{\partial C}{\partial x}\frac{\partial T}{\partial x} \end{array}\right). \end{array}$$

The entropy generation in the above equations consists of these effects:

1. Diffusive irreversibility (DI) (also known as Diffusion).
2. Fluid friction irreversibility (FFI).
3. Heat transfer irreversibility(HTI) (also known as Conduction effect).

The characteristics of entropy generation is

(58) $$S_{0}^{\prime \prime \prime }=\frac{k{\left(\Delta T\right)}^{2}}{L^{2}T_{0}^{2}}.$$

Using Equation (20), the dimensionless form of entropy generation is

(59) $$\begin{array}{c} N_{G}=\frac{S_{gen}^{\prime \prime \prime }}{S_{0}^{\prime \prime \prime }}=Re\left(1+Rd\right)\theta^{\prime 2}\left(\zeta \right)+\frac{ReB_{r}}{\Omega }\left[\begin{array}{c} \left(1+\gamma \right)f^{\prime \prime 2}\left(\zeta \right)-\frac{\gamma \beta }{3}f^{\prime \prime 4}\left(\zeta \right)+Mf^{\prime 2}\left(\zeta \right) \end{array}\right]+\\ Re\lambda_{1}{\left(\begin{array}{c} \frac{\chi }{\Omega } \end{array}\right)}^{2}\phi^{\prime 2}\left(\zeta \right)+Re\lambda_{1}\left(\begin{array}{c} \frac{\chi }{\Omega } \end{array}\right)\theta^{\prime }\left(\zeta \right)\phi^{\prime }\left(\zeta \right). \end{array}$$

These numbers are given in the following form:

(60) $$Re=\frac{{\hat{u}}_{L}L^{2}}{v},B_{r}=\frac{\mu {\hat{u}}_{w}^{2}}{k\Delta T^{\prime }},\Omega =\frac{\Delta T}{T_{0}},\chi =\frac{\Delta C}{C_{0}},\lambda_{1}=\frac{RDC_{0}}{k}.$$

## 7. Convergence of Solution

**Figure 1:**
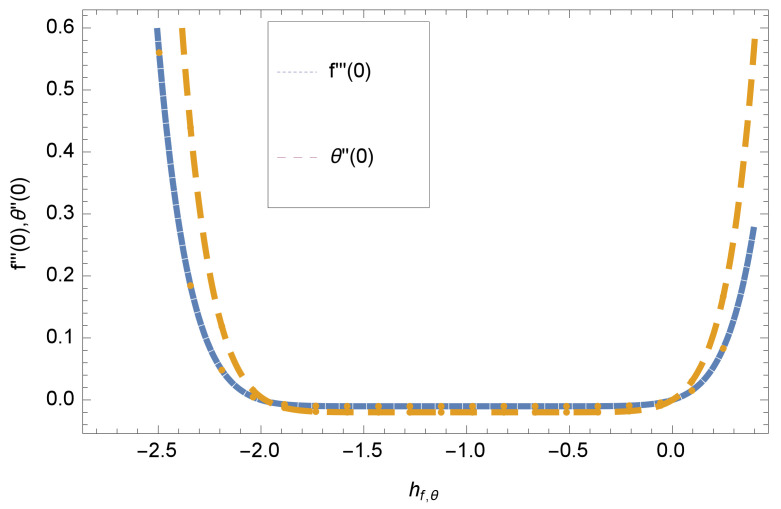
Combine h curve of function f(η) and θ(η) at 7th order approximation, when γ=Sc=A=ξ=β=k=0.1, Nb=Nt=Rd=0.3, M=Pr=1.

**Figure 2:**
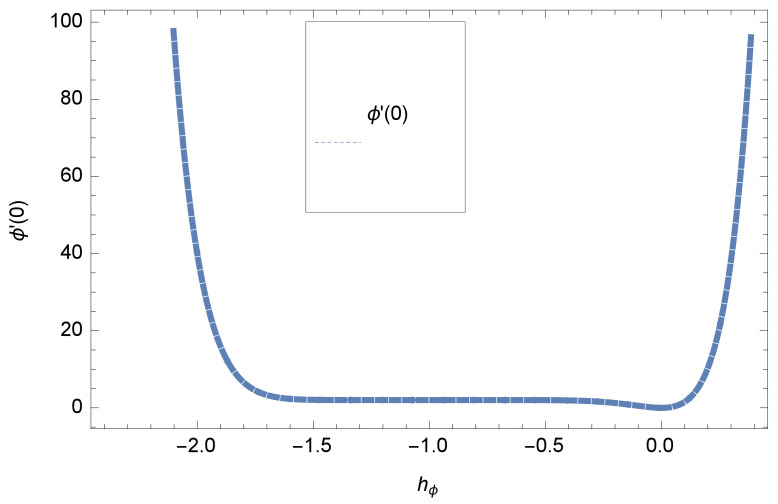
h curve of function ϕ(η) at 7th order approximation, when γ=Sc=A=ξ=β=k=0.1, Nb=Nt=Rd=0.3, M=Pr=1.

When we computed the series solutions of concentration, velocity and temperature functions using HAM, the assisting parameters $h_{f,\theta }$ and $h_{\phi }$ appear, which are responsible for adjusting to converge the solutions. h-curve graphs of $f^{\prime \prime }\left(0\right)$,$\theta^{\prime }\left(0\right)$ and $\phi^{\prime }\left(0\right)$ for various order Approximation are plotted to get the possible region of h curves in Figure 1 and Figure 2 for various values of embedded variables. The h-curves consecutively display the valid region. The convergence region of the h-curve in Figure 1 and Figure 2 is shown in the domain −0.2≤h≤0.0, which is a valid region.

## 8. Results and Discussion

**Figure 3:**
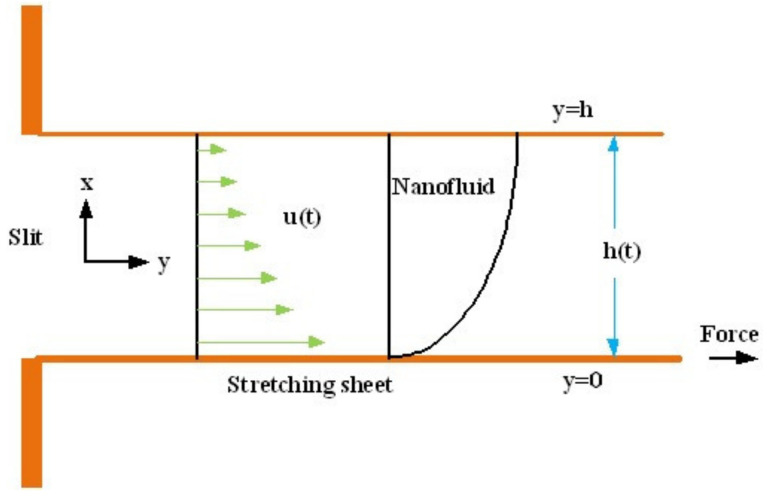
Geometry of the model.

**Figure 4:**
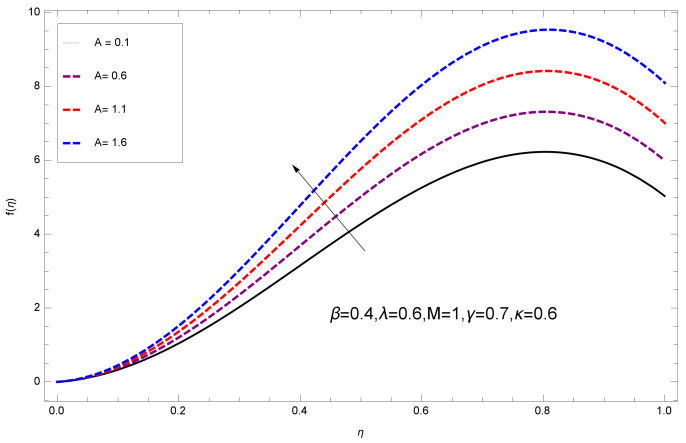
Impact of A on f(η).

**Figure 5:**
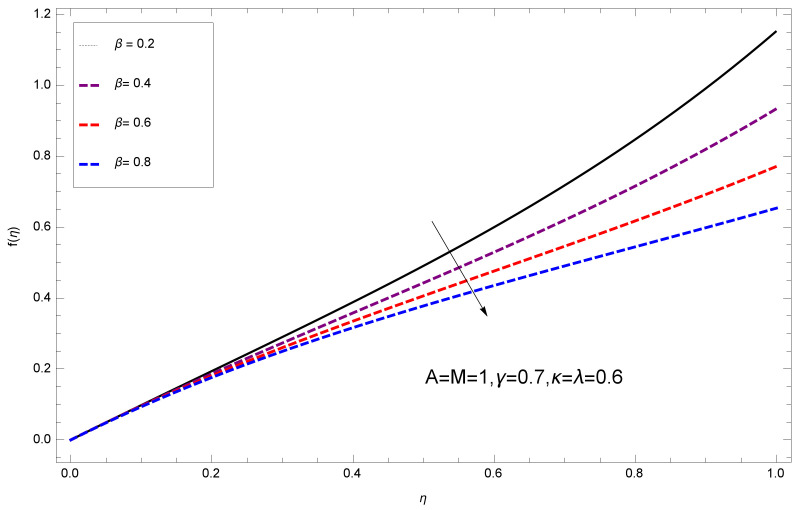
Impact of β on f(η).

**Figure 6:**
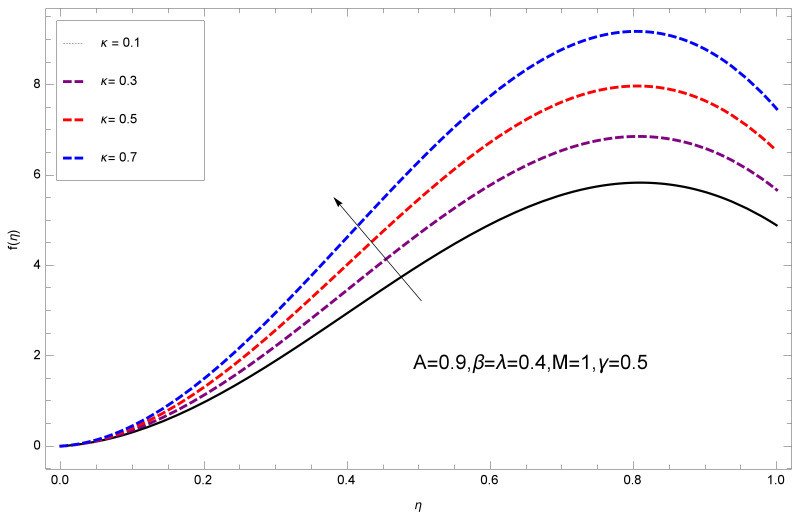
Impact of *k* on f(η).

**Figure 7:**
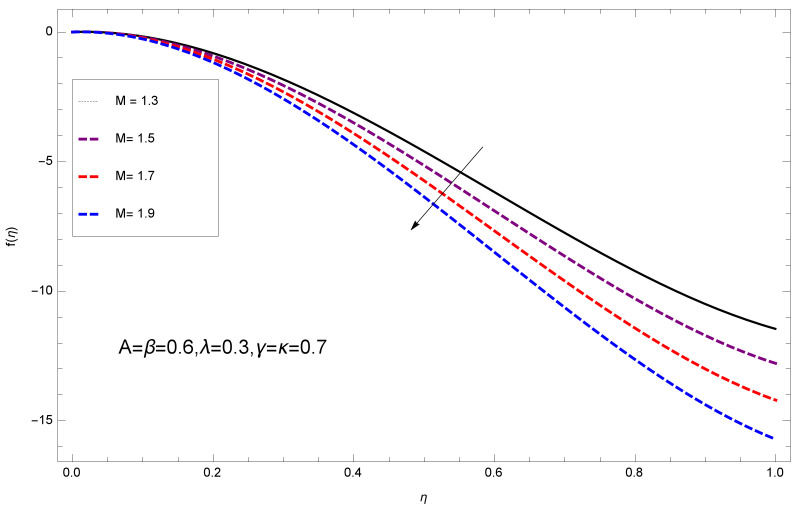
Impact of M on f(η).

**Figure 8:**
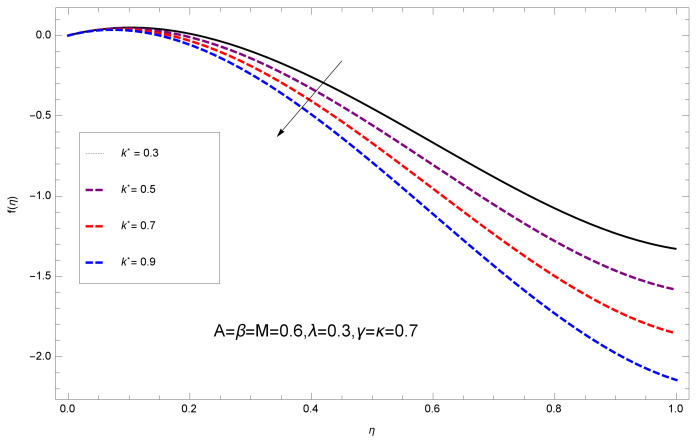
Impact of $k^{*}$ on f(η).

**Figure 9:**
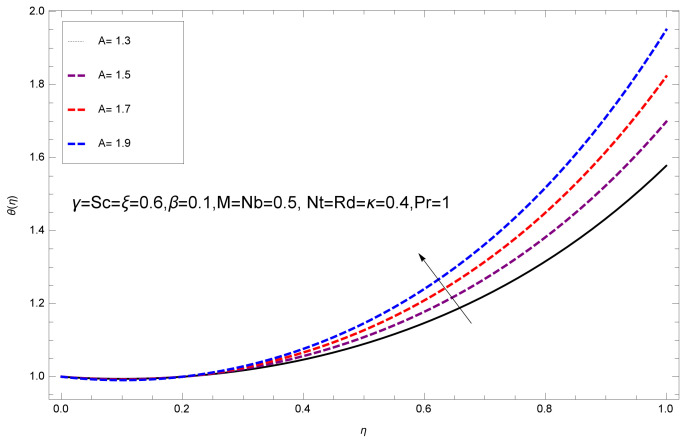
Impact of A on θ(η).

**Figure 10:**
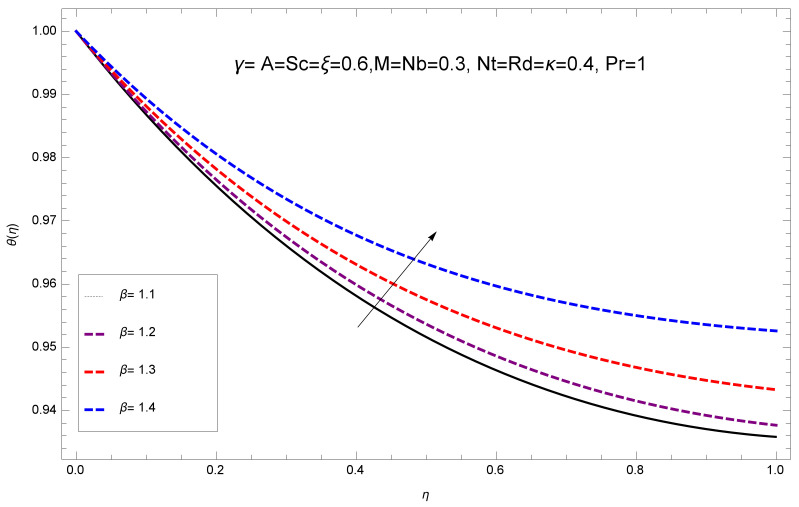
Impact of β on θ(η).

**Figure 11:**
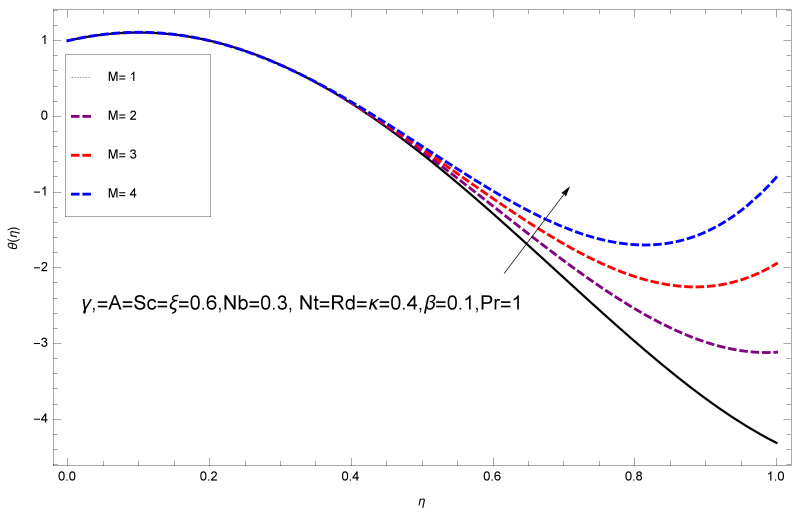
Impact of M on θ(η).

**Figure 12:**
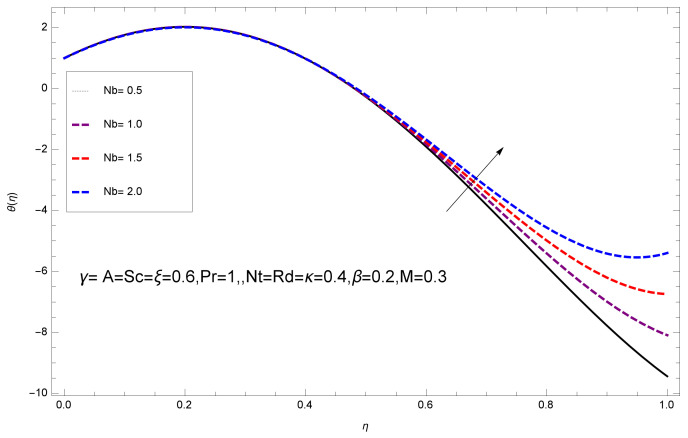
Impact of Nb on θ(η).

**Figure 13:**
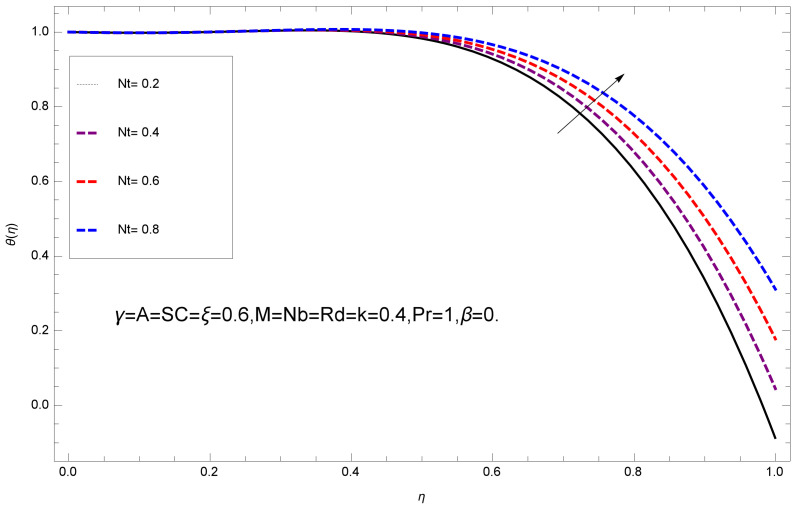
Impact of Nt on θ(η).

**Figure 14:**
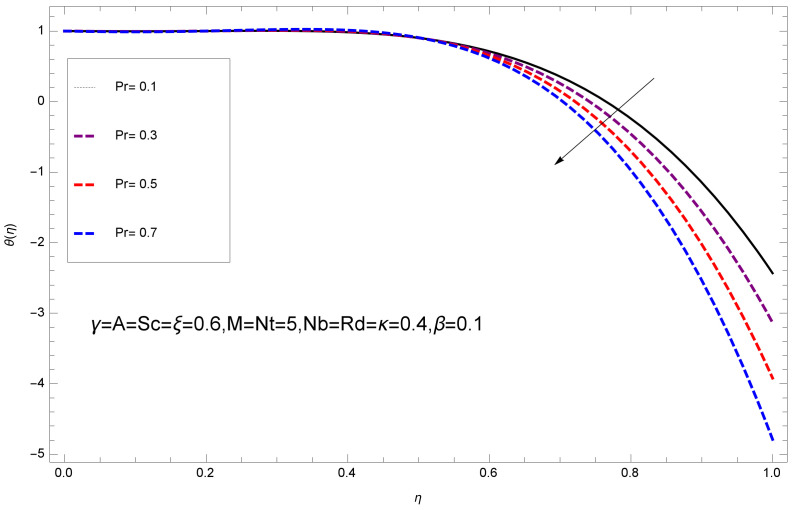
Impact of Pr on θ(η).

**Figure 15:**
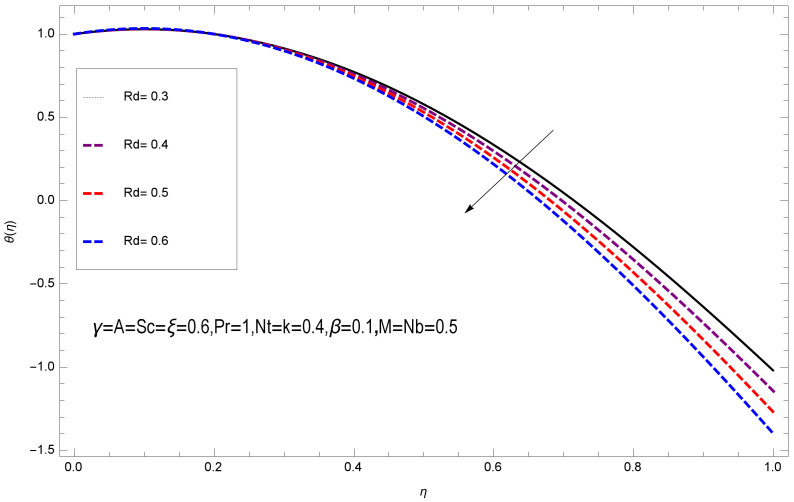
Impact of Rd on θ(η).

**Figure 16:**
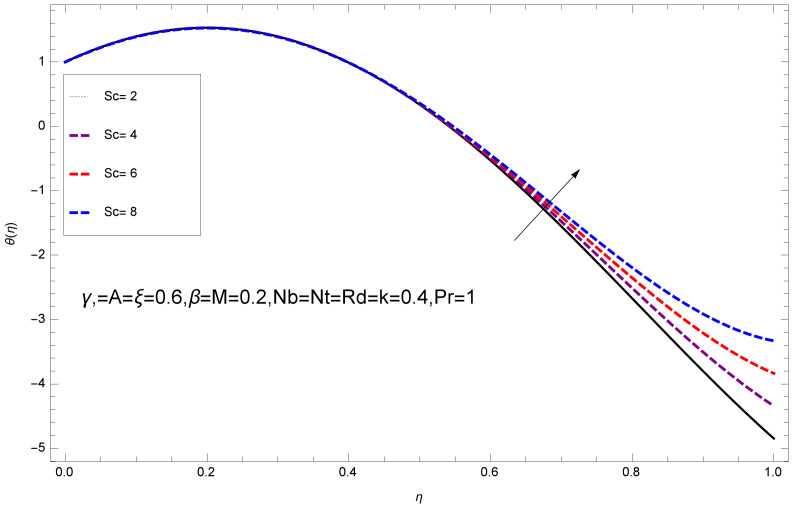
Impact of Sc on θ(η).

**Figure 17:**
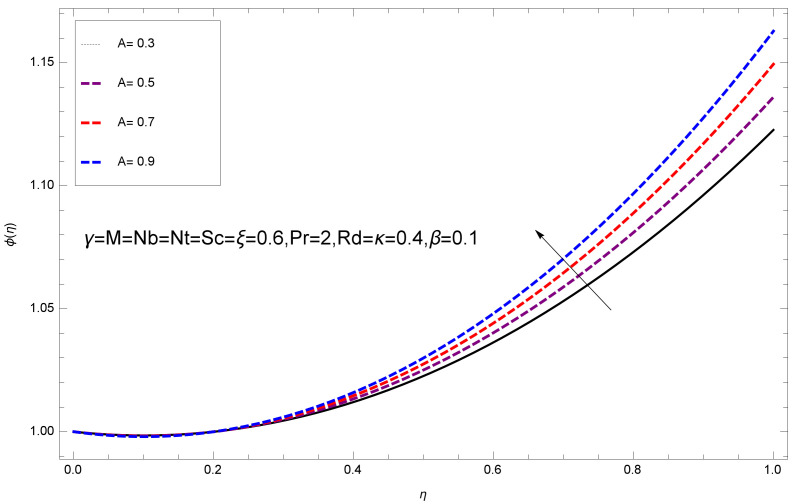
Impact of A on ϕ(η).

**Figure 18:**
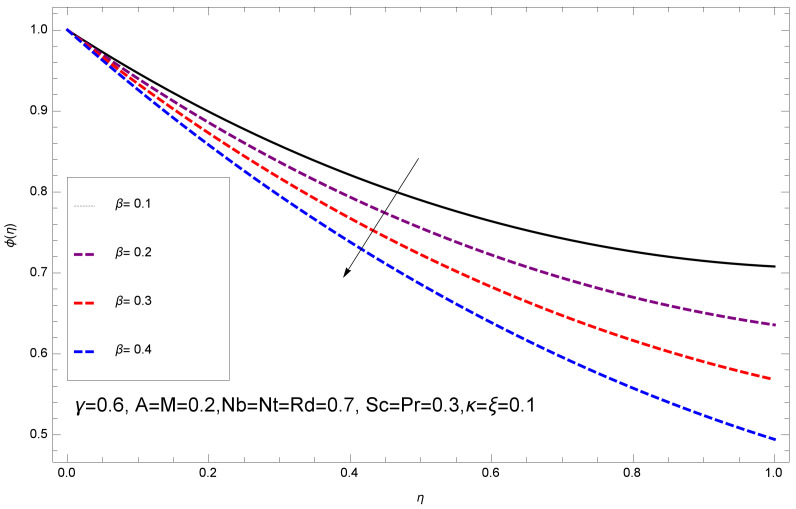
Impact of β on ϕ(η).

**Figure 19:**
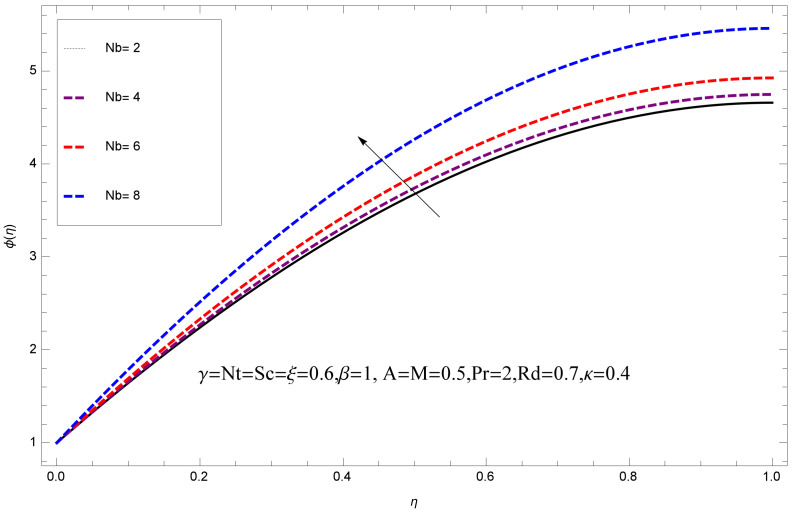
Impact of Nb on ϕ(η).

**Figure 20:**
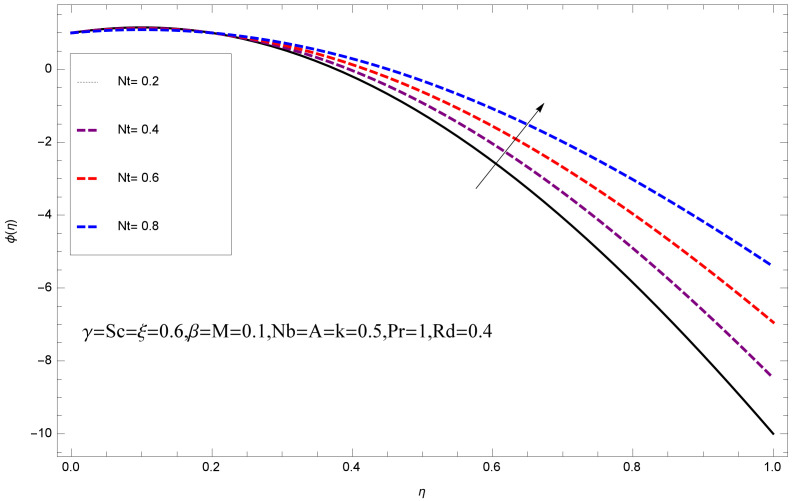
Impact of Nt on ϕ(η).

**Figure 21:**
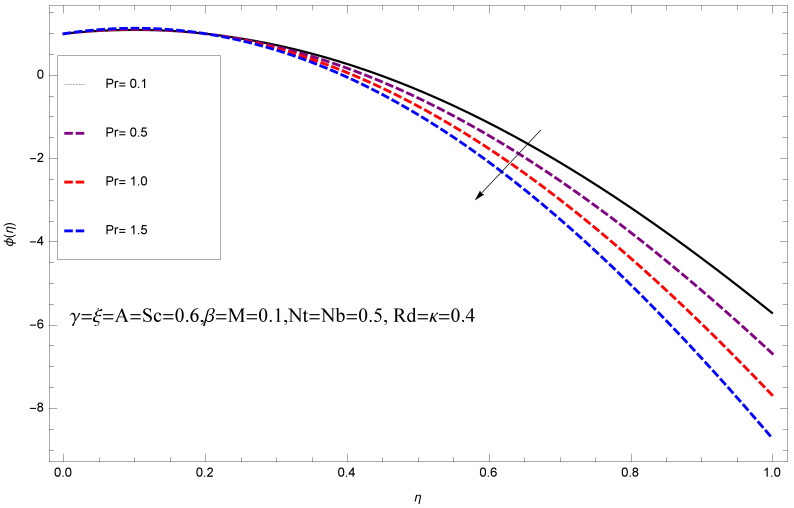
Impact of Pr on ϕ(η).

**Figure 22:**
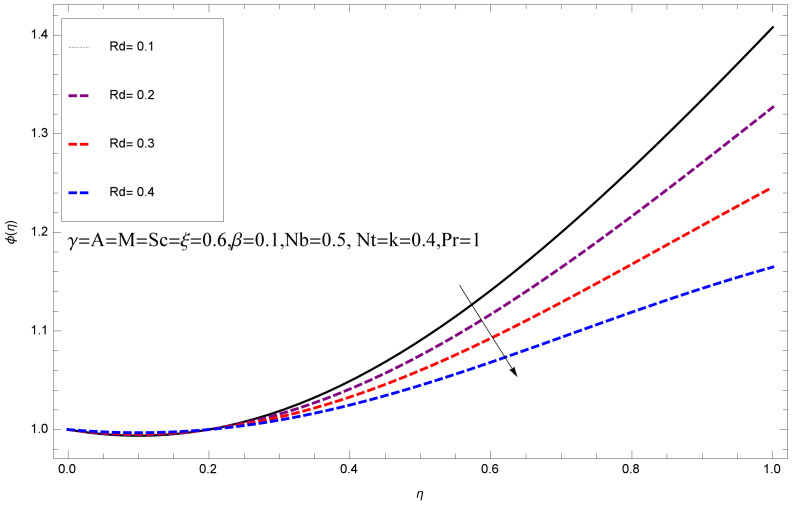
Impact of Rd on ϕ(η).

**Figure 23:**
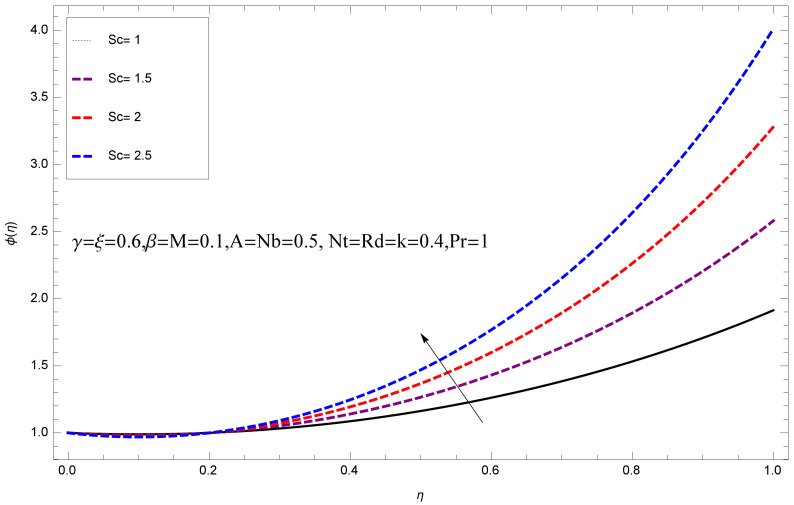
Impact of Sc on ϕ(η).

**Figure 24:**
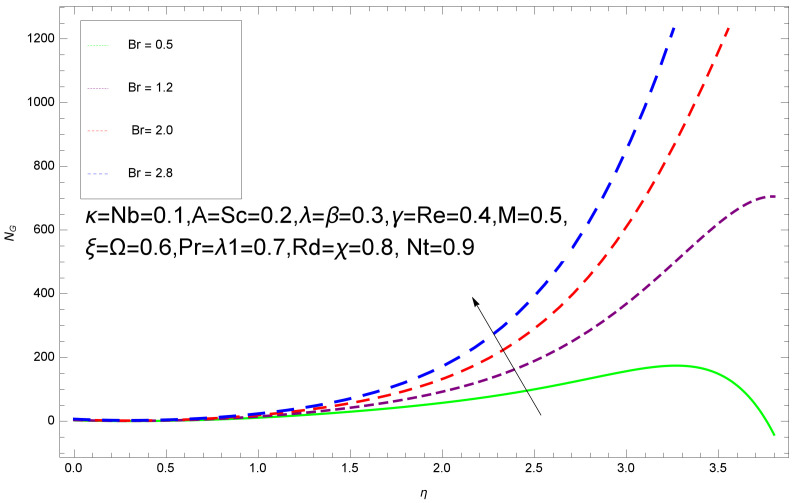
Entropy profile for various values of Br.

**Figure 25:**
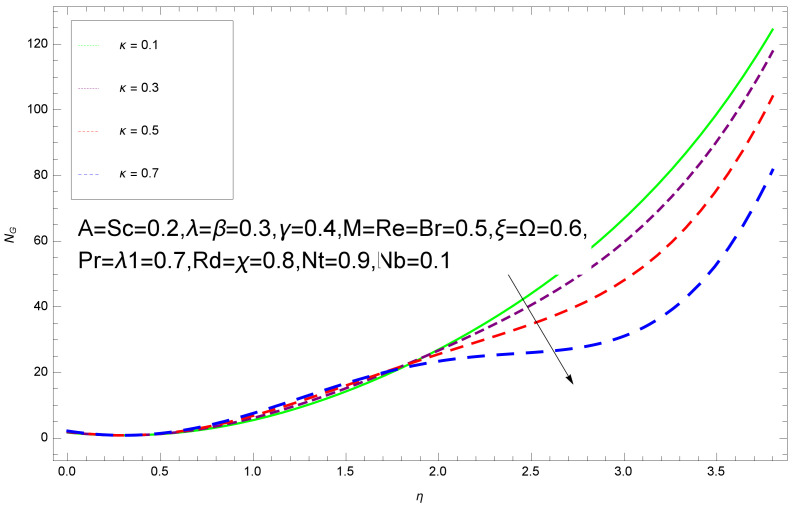
Entropy profile for various values of k.

**Figure 26:**
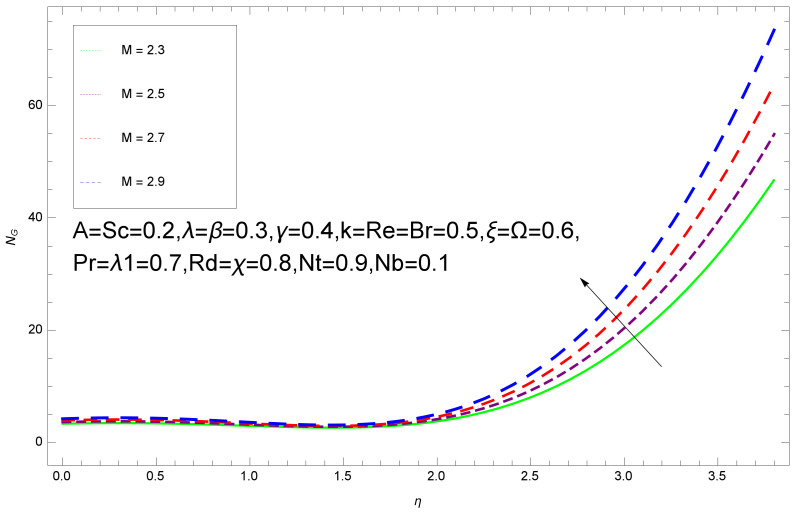
Entropy profile for various values of M.

**Figure 27:**
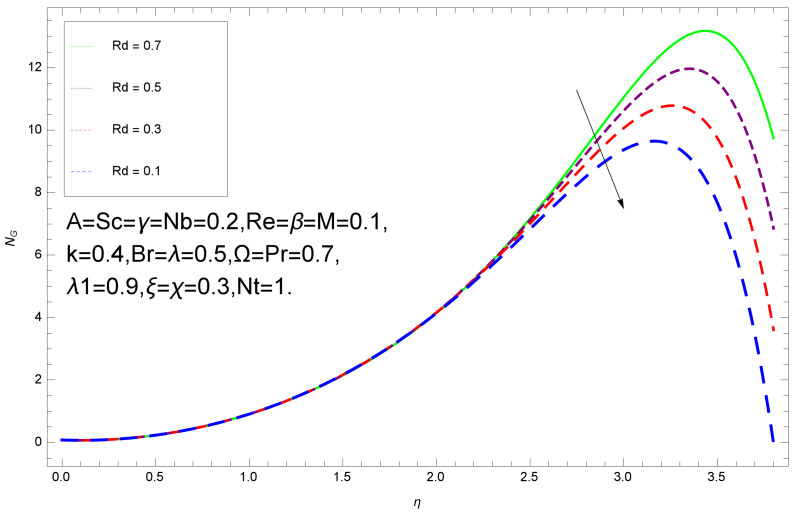
Entropy profile for various values of Rd.

**Figure 28:**
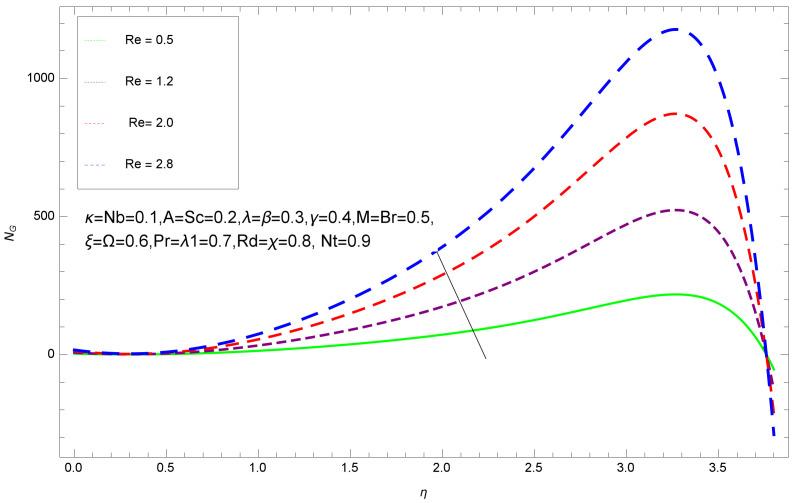
Entropy profile for various values of Re.

This section deals with the theoretical and graphical behavior of different physical quantities that are obtained in the present flow problem. The computational software Mathematica has been utilized to investigate the novelties of all the physical parameters. Figure 3 shows the physical model of the problem. The graphs of h-curve for different order Approximation are plotted in Figure 1 and Figure 2 for various values of embedded variables. The h-curves consecutively display the valid region. In particular, we discuss the influence of various embedded parameters on velocity profile, temperature profile, nanoparticle concentration profile, and entropy profile. The graphical explanation of these parameters has been displayed in figures [4,5,6,7,8,9,10,11,12,13,14,15,16,17,18,19,20,21,22,23,24,25,26,27,28]. The influence of unsteady constraint *A* on the f(η) profile illustrated in Figure 4. The velocity field f(η) rises with the rise in unsteady parameter *A*. The effect of film thickness β has been demonstrated for various values of fluid velocity mentioned in Figure 5. It is seen that f(η) falls over with higher values of β.The impact of Erying fluid factor *k* over the f(η) is exposed in Figure 6. It has been observed that, when Erying fluid parameter *k* increases, then it raises the nanofluid film motion, and this effect is clear at the stretching surface. The characteristics of magnetic factor *M* on fluid velocity profile is shown in Figure 7. It is obvious from mathematical formulation that the magnetic parameter *M* is inversely varied with velocity distribution f(η). Increasing magnetic parameter *M* decreases the velocity field. This influence of magnetic field is caused by the production of friction force to the movement known as the Lorentz force, which brings retardation to the flow of the fluid and hence reduces fluid velocity at the edge.The characteristics of porosity parameter $k^{*}$ on velocity field is shown in Figure 8, which have an imperative character in the flow motion. Increasing $k^{*}$ increases the porous space which creates resistance in the flow path and reduces the flow motion. In fact, growing values of $k^{*}$ show the large number of porous spaces, which create resistance in the flow path and reduce overall fluid motion. Basically, with the increaseing number of holes in the porous plates the nanoliquid particles face hurdles in flow over these holes. Throughout this motion the way is not clear and the fluid has to decrease its velocity at any point. The unsteady parameter *A* has an opposite effect on temperature profile. Figure 9 depicts that temperature depreciates with the unsteady parameter *A*. Each and every fluid has the similar effect on temperature for the unsteady parameter *A*. The fluid produces confrontation to the flow of film and shows a tendency to reduce the velocity of fluid flow having larger values of β and it is clear in Figure 10. The fluid film size absorbs heat that causes fall down in temperature distribution. The characteristics of magnetic factor *M* on temperature profile is shown in Figure 11. The steepness in the temperature profiles decreases with decreasing the width of the thermal boundary layers. The free surface temperature is increased with the Brownian motion constraint as illustrated in Figure 12. Actually, Brownian motion is the erratic random movement of microscopic particles in a fluid, as a result of continuous bombardment from molecules of the surrounding medium. The reality is that arbitrary motion of particles of the fluid generates collision in the particles. Increase in the value of Brownian motion constraint Nb results in an increase in temperature of the fluid. Consequently, it causes reduction in free surface nanoparticle volume fraction. Due to kinetic molecular theory, the heat of the fluid increases due to the increase of Brownian motion. Thus, the given result is in good agreement with the real situation. The thermophoresis parameter Nt faces depreciation in contrast with temperature profile. This phenomenon is described by Figure 13. The thermophoresis limitation supports growing the surface temperature. The irregular moment of nano suspended particles in the fluid represented the Brownian motion. Due to this irregularity in motion, nano suspended particles produce kinetic energy and the temperature increases; as a result, the thermophoretic force is initiated. This force causes intensity in the fluid to move away from the surface of the stretching sheet. Subsequently, the temperature inside the boundary layer rises as Nt grows.Physically, Prandtl number is the ratio of kinematic viscidness to thermal diffusivity and is a dimensionless quantity. The Pr is increased when the value of momentum diffusivity is greater than the thermal diffusivity. Thus, heat transmission at the surface grows with the increase in Pr values while mass transmission is concentrated as the Prandtl number grows. The impact of Pr is given in the Figure 14. It clearly shows that θ(η) reduces with large Pr number. The logic behind this is that, with the large value of Pr, thermal layer of the boundary reduces. The consequence is more noticeable for slight Prandtl quantity as the width of the thermal boundary layer is relatively greater. The influence of Rd parameter on temperature is presented in Figure 15. Thermal radiation has an imperative part in inclusive surface heat transmission when the coefficient of convection heat transmission is small. When we increase the value of Rd, it is perceived that it augments the heat in the boundary layer of the fluid. This increase causes a drop in the rate of cooling in nanofluid flow. The heat field θ(η) increases with the change in the Schmidt number illustrated in Figure 16. It is obvious that the flow part increases in the horizontal direction by giving rise in the Schmidt number. It is trivial that, with a rise in the Schmidt number, the flow part increases in the *x*-direction. The logic behind is that the Schmidt parameter is the ratio of momentum and concentration diffusivities. The rise in the values of Sc decreases width of the fluid and causes fall down in θ(η). It is obvious from Figure 17 that the increasing values of unsteady parameter A increases the concentration profile ϕ(η). The concentration of the fluid ϕ(η) rises as values of β progress, as exhibited by Figure 18. The logic behind this is that the fluid film width exhibits a direct relation with thermal conductivity and viscosity. The impact of varying Nb parameter with respect to the concentration profile ϕ(η) on domain 0≤η≤1 has an increasing impact of ϕ(η) and has been observed for both suction and injection, and it is displayed in Figure 19. As thermophoresis parameter Nt rises, elevation occurs in the concentration profile. Thermophoresis restriction also helps in rising the surface nano particle volume fraction like the surface temperature shown in Figure 20. The surface mass transfer rate in steady and unsteady cases decreases with increasing the thermophoresis factor Nt, but show high surface mass transfer rate in unsteady cases as compared to steady ones. Concentration profile exhibits the inverse relation with Pr number shown in Figure 21. It means that thinning of the thermal boundary layer progresses the flow in the *x*-direction, which is reflected in the graph. The influence of Rd parameter on concentration profile is presented in Figure 22. When we increase the value of Rd, it is perceived that it augments the concentration in the boundary layer of the fluid. This increase causes a drop in the rate of cooling in nanofluid flow. The non-dimensional concentration profile reduces with dissimilar measures of parameter Sc shown in Figure 23. It is obvious that a flow part increases in the horizontal direction by giving rise in the Schmidt number. It is trivial that, with a rise in the Schmidt number, the flow part increases in the *x*-direction. The logic behind is that the Schmidt parameter is the ratio of momentum and concentration diffusivities. The viscidness dissipation effect on the nanoparticle volume fraction is insignificant for higher quantities of Schmidt numbers. Figure 24, Figure 25, Figure 26, Figure 27 and Figure 28 represent the entropy profile for the Brinkmann Br, Eyring–Powell parameter *k*, Magnetic parameter *M*, Radiation parameter Rd and Reynolds number Re. It is clear from Figure 24, Figure 26 and Figure 28 that the entropy profile increases due to increase in Br, *M*, and Re, respectively. On the other hand, it is reflected from Figure 25 and Figure 27 that the entropy generation field decreases with increasing values of parameter *k* and Rd.

## 9. Tables Discussion

Table quantities such as film thickness β, skin friction co-efficient $f^{\prime \prime }\left(0\right)$, heat flux $Nu=-\theta^{\prime }\left(0\right)$ and mass flux $Sh=-\phi^{\prime }\left(0\right)$ for engineering interest are calculated through Table 1, Table 2, Table 3, Table 4 and Table 5. In Table 1, values of thin film thickness β are determined using increasing value of A. It is analyzed that thin film thickness reduces randomly when A is increased. The present results are compared with Wang [3], Narayana and Sibanda [39], and Qasim [15]. Complete agreement has been found among all the results. Table 2 compares the present results of skin friction co-efficient with Wang [3], Narayana and Sibanda [39], and Qasim [15] for increasing values of S using M, A, β and γ closed agreement has been found. In Table 3, the effect of M, Nt, A and Pr on wall temperature is calculated taking A=0.8. The large value of M and Nt increases the wall temperature while the large value of A and Pr reduces the wall temperature. Table 4 examines the impacts of embedding parameters Nb, β, Pr and Nt on the heat flux $Nu=-\theta^{\prime }\left(0\right)$ and mass flux $Sh=-\phi^{\prime }\left(0\right)$. It has been seen that the increasing values of Nb, β and Pr decrease mass flux while the increasing values of Nt increases mass flux. It has also been seen that the increasing values of embedded parameters randomly varies heat flux. The current results of $-\theta^{\prime }\left(0\right)$ and $-\phi^{\prime }\left(0\right)$ has also been compared with the previous one. The present outcomes are compared with the foregoing ones, and an outstanding agreement is initiated between the current and previous results. The results obtained for velocity, temperature and concentration profiles through HAM have also been weighted up in the tables [5], respectively, which clearly reflects the convergence of the homotopy analysis method.

## 10. Conclusions

The present research work investigates entropy generation in two-dimensional nanofluid film flow of Eyring–Powell Fluid along with variable heat transmission through a porous stretching surface in the existence of constant magnetic field (MHD). The flow of liquid films are taken under the consideration of thermal radiation. The observation of this work depends upon the impact of variable temperature, thermal radiation and magnetic field on nanoliquid film flows. The modeled equations are solved analytically using HAM. The convergence has been shown numerically and the effect of the embedded parameters are observed and studied graphically. The influence of the skin fraction and nusslet number and sherwood number is shown numerically.

The central concluded points are as:

- It is perceived that the large values of Magnetic parameter *M* drop the velocity distribution of the nanofluid films.
- Entropy profile increases with the increasing values of Brinkmann Br, Hartmann number *M*, and Reynolds number Re.
- Entropy profile decreases with the increasing values of Eyring–Powell parameter *k* and Radiation parameter Rd.
- The larger values of Brownian motion Nb parameter raises the profile of temperature.
- The thermal boundary layer thickness reduces with rise of Sc and Nusselt number rises with rise in Radiation parameter.
- The growing behavior of Pr increases the surface temperature, where the opposite effect is found for an unsteady parameter *A*, that is, the large values of *A* reduce the surface temperature.
- Porosity parameter decreases the motion of the liquid films.
- It is perceived that the Thermal Radiation parameter Rd decreases temperature profile, when it is increased and the same effect is observed for the concentration field.
- It is observed that the heat profile falls with the large values of thermophoresis parameter Nt and increases for small values.
- The increasing values of Nb reduce the mass flux, where Nt increases the mass flux. The higher values of Re reduce the mass flux, while it rises with rising values of Sc.
- The convergence of the HAM method with the variation of the physical parameters observed numerically.
- Non-dimensional velocity reduces with variable viscosity and magnetic constraints.
- The temperature gradient and concentration fields are both directly related with magnetic field.
- Rise in the nanoparticle concentration efficiently increases the friction feature of Eyring–Powell nanofluid.

## Figures and Tables

**Table 1:** Comparision of values of film thickness β for various values of A.

A	Wang [3]	Narayana and Sibanda [39]	Qasim [15]	Present Results
	β	β	β	β
0.4	5.122490	4.981455	4.981454	5.523451
0.6	3.131250	3.131713	3.131710	4.002111
0.8	2.151990	2.151994	2.151994	3.992358
1.0	1.543620	1.543618	1.543616	3.113001
1.2	1.127780	1.127780	1.127781	1.625391
1.4	0.821032	0.821032	0.821032	1.896541
1.6	0.576173	0.576173	0.576173	0.876512
1.8	0.356389	0.356389	0.356389	0.266156

**Table 2:** Comparision of values of skin friction coefficient $f^{\prime \prime }\left(0\right)$ and $\beta f^{\prime \prime }\left(0\right)$ for various values of A.

A	Wang [3]	Narayana and Sibanda [39]	Qasim [15]	Present Results
	$f^{\prime \prime }\left(0\right)$	$\beta f^{\prime \prime }\left(0\right)$	$\beta f^{\prime \prime }\left(0\right)$	$\beta f^{\prime \prime }\left(0\right)$
0.4	−6.699120	−5.6494483	−5.6494474	−4.33027
0.6	−3.742330	−3.7427896	−3.7427863	−3.94882
0.8	−2.680940	−2.6809660	−2.6809656	−2.64208
01	−1.972380	−1.9723877	−1.9723819	−1.33999
1.2	−1.442631	−1.4426237	−1.4426252	−0.92157
1.4	−1.012784	−1.0127798	−1.0127802	−0.56897
1.6	−0.642397	−0.6423970	−0.6423970	−0.34227
1.8	−0.309137	−0.3091369	−0.3091367	−0.03027

**Table 3:** The wall temperature for dissimilar values of M, A, Pr and Nt when A=0.8, Nb=0.4, β=0.9, Nb=1.6.

M	Nt	A	Pr	Tawade et al. (2016) Results	Qasim et al. (2016) Results	Present (2017) Results
				θ(β)	θ(β)	θ(β)
0	0.1	1.0	0.1	0.257696	0.9604803	0.223456
1				0.420739	0.6925326	0.432111
2	0.01			0.526782	0.0978841	0.712351
	0.1	0.0		0.695757	0.0248625	1.023001
		1.0	0.1	1.030899	0.0083111	1.625341
			0.2	0.931433	0.0013612	1.236540

**Table 4:** The Nusslet number $\Theta^{\prime }\left(0\right)$ and Sherwood numbers $\Phi^{\prime }\left(0\right)$ versus various values of embedded parameters when A=0.8.

Ec	*β*	Pr	Nt	−Θ^′^(0)	−Θ^′^(0)	−Φ^′^(0)	−Φ^′^(0)
				Tawad et al. (2016) Results	Present (2017) Results	Qasim et al. (2016) Results	Present (2017) Results
0.0	0.2	1.0	0.1	2.46682	0.682385	4.69946	6.68238
0.5				1.66004	0.541422	5.63125	4.94142
1.0				1.17173	0.440569	5.73992	5.44569
	0.2			2.08356	0.321022	4.96867	5.12101
	0.3			1.37004	0.300420	5.68398	5.70742
	0.4			0.94740	0.291420	5.75820	5.29140
		0.5		2.46062	0.371420	4.65665	5.37143
		1.5		1.65905	0.182285	5.59404	6.78223
		5.0		1.17298	0.011422	5.70473	7.01147
			0.4	1.96299	0.612427	5.01443	4.11207
			0.6	1.28112	0.691428	5.66638	4.69458
			0.8	0.87980	0.500987	5.73093	7.50097

**Table 5:** Convergence of $f^{\prime \prime }\left(0\right)$, $\theta^{\prime }\left(0\right)$ and $\phi^{\prime }\left(0\right)$ by HAM when Nt=Nb=Rd=0.3, A=Sc=k=ξ=γ=β=0.1, Pr=M=1.

Solution Approximation	*f*^′′^(0)	*θ*^′^(0)	*ϕ*^′^(0)
1	−0.05401	−0.10070	−1.10075
4	−0.10218	−0.18890	−1.38506
7	−0.10813	−0.19903	−1.88867
10	−0.10888	−0.20281	−1.99293
13	−0.10894	−0.20154	−2.01113
14	−0.10896	−0.20475	−2.01406
17	−0.10897	−0.20478	−2.01451
20	−0.10897	−0.20479	−2.01458
25	−0.10897	−0.20479	−2.01458

## Abbreviations

The following abbreviations are used in this manuscript:

Sh	Sherhood number
Nu	Nusslet number
Re	Reynold number
Pr	Prandtl number
Sc	Schmidth number
$U_{0}\left(x,t\right)$	Stretching velocity
$C_{ref}$	Reference concentration
$T_{ref}$	Reference temperature
*T*	Cauchy stress tensor
$\tau_{ij}$	Extra stress tensor
υ	Kinematic viscosity
μ	Dynamic viscosity
*T*	Temperature
$B_{0}$	Magnetic field strength
$k_{p}$	Thermal conductivity
$q_{r}$	Radioactive heat fluctuation
ϕ	Stefan Boltzmann constant
Ψ(x,y,t)	Stream function
$k^{*}$	Porosity parameter
Br	Brinkmann number
β	Film Thickness parameter
*A*	Unsteady parameter
*M*	Magnetic parameter
ξ	Stretching parameter
$D_{B}$	Brownian diffusion of nanofluids
Nt	Thermophoretic parameter
Nb	Brownian motion parameter
Rd	Rediation parameter
γ	Stretching parameter
*I*	Identity tensor
ρ	Density
σ	Electrical conductivity
α	Thermal diffusivity
$c_{p}$	Specific heat
$D_{B}$	Brownian diffusion
δ(t)	Thickness of liquid
κ	Absorption coefficient
λ	Stretching parameter
*k*	Eyring–Powell fluid parameter
